# Closed-Surface Multifunctional Antireflective Coating Made from SiO_2_ with TiO_2_ Nanocomposites

**DOI:** 10.3390/ma14061367

**Published:** 2021-03-11

**Authors:** Zhiqiu Guo, Ze Zhu, Ya Liu, Changjun Wu, Hao Tu, Jianhua Wang, Xuping Su

**Affiliations:** 1 Jiangsu Collaborative Innovation Center of Photovoltaic Science and Engineering, Changzhou University, Changzhou 213164, China; zhiqiu.guo@jinkosolar.com (Z.G.); b20080514@smail.cczu.edu.cn (Z.Z.); yliu@cczu.edu.cn (Y.L.); wucj@cczu.edu.cn (C.W.); tuhao@cczu.edu.cn (H.T.); wangjh@cczu.edu.cn (J.W.); 2 Jinko Solar Co., Ltd., Jiaxing 314416, China; 3 Jiangsu Key Laboratory of Materials Surface Science and Technology, Changzhou University, Changzhou 213164, China

**Keywords:** antireflective coating, self-cleaning, closed-surface, one-dipping method, SiO_2_-TiO_2_

## Abstract

An SiO_2_-TiO_2_ closed-surface antireflective coating was fabricated by the one-dipping method. TiO_2_ nanoparticles were mixed with a nanocomposited silica sol, which was composed of acid-catalyzed nanosilica networks and silica hollow nanospheres (HNs). The microstructure of the sol-gel was characterized by transmission electron microscopy. The silica HNs were approximately 40–50 nm in diameter with a shell thickness of approximately 8–10 nm. The branched-chain structure resulting from acidic hydrolysis grew on these silica HNs, and TiO_2_ was distributed inside this network. The surface morphology of the coating was measured by field emission scanning electron microscopy and atomic force microscopy. After optimization, transmittance of up to 94.03% was obtained on photovoltaic (PV) glass with a single side coated by this antireflective coating, whose refractive index was around 1.30. The short-circuit current gain of PV module was around 2.14–2.32%, as shown by the current-voltage (IV) curve measurements and external quantum efficiency (EQE) tests. This thin film also exhibited high photocatalytic activity. Due to the lack of voids on its surface, the antireflective coating in this study possessed excellent long-term reliability and robustness in both high-moisture and high-temperature environments. Combined with its self-cleaning function, this antireflective coating has great potential to be implemented in windows and photovoltaic modules.

## 1. Introduction

Due to differences in the refractive index (RI), light is always reflected when it travels from one medium to another, and the higher the difference, the greater the reflectance and vice versa. An antireflective coating (ARC) with a RI between these two mediums could reduce light reflection. For the interface between air and substrate, minimum reflection of a single-layer ARC can be achieved when the RI of the coating is equal to the square root of the substrate’s RI [1]. Specifically regarding photovoltaic modules and building windows, since the RI of air-side glass is around 1.52, the target RI of the ARC should be around 1.23, which is lower than the RI of SiO_2_ and TiO_2_ [2]. According to Yang et al., pores in nano thin films could reduce their RI [3], so ARCs are typically made of porous nanoparticles, such as SiO_2_ nanoparticles and TiO_2_ nanoparticles [4,5].

For silica ARCs on PV glass, porosity of 40% is the minimum requirement in the coating to achieve the target RI [6]. Porous silica ARCs made from nanoparticles contain not only pores in the coating but also many voids on the surface; they easily absorb moisture and organic contaminants from the air, resulting in large variations in the RI [7,8]. In addition, silica nanoparticles inside thin film are connected to each other, as well as to the glass substrate only by van der Waals forces, which result in ARCs with poor mechanical properties and poor durability [9]. To overcome these issues, many studies have designed and fabricated closed-surface silica ARCs with good robustness and reliability. These studies can be sorted into three groups: (1) Infiltrating stronger materials into a porous, thin film. By infiltrating zeolite, Chen et al. [10] increased the intended hardness of an amorphous silica ARC to 1.5 GPa in addition to an elastic modulus of up to 35 GPa. Zhang et al. [11] reported the infiltration of acid-catalyzed silica with a branched-chain structure into the voids of an ARC to prepare closed-surface ARCs on PV glass, which showed a maximum transmittance of up to 96.86–97.34%. (2) Growing multi-layer ARCs layer by layer (LBL). In the study conducted by Cohen et al., both open-surface and closed-surface silica ARCs were fabricated by LBL dipping [12], but ARCs from this study suffered a significant transmission reduction in the wavelength range from visible to near-infrared regions. (3) A simple one-dipping method, which has been developed recently. Guo et al. [13] reported a one-dipping sol-gel method for silica hollow nanospheres (HNs) and acid-catalyzed silica. The acid-catalyzed silica with a branched-chain structure cross-linked silica HNs to silica HNs, as well as to the glass substrate, to improve the robustness of the thin film. This acid-catalyzed silica also blocked voids on the front surface of the ARC to prevent moisture from penetrating the front surface to attack the Si-O-Si network in the thin film. By optimizing the ratio of silica HNs to acid-catalyzed silica, a weather-resistant closed-surface ARC with a maximum transmittance of 96.92% was obtained.

Self-cleaning is a premium function of ARCs, especially those used in outdoor applications, but ARCs made of pure silica do not reveal this self-cleaning property. Titanium dioxide (TiO_2_) is one of the most interesting semiconducting metal oxides for photocatalysis [14], and its wide bandgap energy (i.e., 3.0–3.2 eV) allows it to absorbs UV light, which generates holes (h) and electrons (e) that can subsequently induce redox reactions to decompose organic contaminants [15,16]. Many works have investigated both TiO_2_ ARCs and hybrid SiO_2_-TiO_2_ ARCs. Kermadi et al. [17] synthesized xTiO_2_(1 − x)SiO_2_ thin films, and by controlling the thin film composition, the RI of the ARC could be tuned from 1.48 to 2.18 at λ = 632.8 nm. Tao et al. [18] reported a broadband TiO_2_-SiO_2_ stack coating via an LBL technique; a maximum transmittance of 96.0% was achieved, and the film surface showed excellent superhydrophilicity (water contact angle ≈ 3.0°) and antifogging properties. Yao et al. [19] prepared an AR sol-gel with SiO_2_-TiO_2_ double-shell HNs in which a TiO_2_ shell layer was grown on the silica HNs. It displayed a maximum transmittance of 99.4%, with an average transmittance of 98.5% in the visible region.

In this work, SiO_2_-TiO_2_ closed-surface ARCs are proposed to have both long-term reliability and photocatalytic functions. A simple one-dipping process is used to prepare this SiO_2_-TiO_2_ closed-surface ARC by adding TiO_2_ nanoparticles into a silica nanocomposite AR thin film. The silica composite sol-gel was first made by growing a branch-chain-structured, acid-catalyzed silica on silica HNs. The branched silica was designed to link silica HNs with each other as well as to the glass substrate. Then, the silica composite sol-gel was mixed with a TiO_2_ sol-gel, and the mixture was coated onto glass by a one-dip process. Organics were removed by subsequent calcination. By optimizing the sol-gel content, a multi-functional closed-surface ARC was obtained with 2.3 wt% TiO_2_. A maximum transmittance of 94.03% was obtained when a single side of PV glass was coated with the ARC to obtain an RI of around 1.30. This ARC showed excellent photocatalytic decomposition activity towards organic contaminants and long-term reliability in accelerated aging tests.

## 2. Materials and Methods 

### 2.1. Materials

Tetrabutyltitanate (TBOT), tetraethylorthosilicate (TEOS, 98%), ammonium hydroxide (NH_4_OH, 8–28%), hydrochloric acid (HCl, 8%), acetic acid (HAC, 98%), absolute ethanol (EtOH, 99.5%), nitric acid (HNO_3_, 68%), and 3-glycidyloxypropyltrimethoxysilane (KH-560, 97%) were purchased from Sinopharm (Beijing, China). Polyacrylic acid (PAA, M.W. ~ 3000 g/mol) was purchased from Wokai (Shanghai, China). All materials were of analytical grade. Deionized (DI) water with a resistivity of 18 MΩ·cm was used in all experiments. PV glass substrates with a thickness of 3.2 mm and a patterned rear side were purchased from Xinyi Co., Ltd. (Wuhu, China).

### 2.2. Preparation of Solutions

PAA (1.50 g) was dissolved in 22 mL aqueous ammonium hydroxide, and the mixture was slowly added to 500 mL of absolute EtOH under vigorous magnetic stirring at room temperature to form 40–50 nm PAA spheres. Five aliquots of TEOS totaling 10 mL subsequently underwent vigorous magnetic stirring at 1 h intervals. After storage at room temperature for 12 h, 8–10 nm silica shells were formed on the PAA spheres. Ammonia in the sol was then removed by refluxing at 80 °C in a ventilating cabinet for 6 h. The sol was then concentrated to a solid content of around 3%.

A total of 12 mL of TEOS, together with H_2_O, HCl, and EtOH, with a molar ratio of TEOS:HCl:H_2_O:EtOH = 1:0.3:15:28, was added into the above sol under vigorous stirring. Then, 3 aliquots of KH-560 totaling 12 mL were injected into the solution at 1 h intervals. The mixed sol was then sealed and aged for 5 d at room temperature to grow silica with a branched-chain structure on PAA-silica HNs.

Two solutions were prepared under stirring separately, one with 35 mL TBOT, 6.5 mL HAC, and 105 mL EtOH, and the other with 70 mL EtOH, 3.6 mL H_2_O, and 1 mL HNO_3_. These two solutions were then mixed slowly under stirring. The combined solution was boiled for 5 min; then, 1000 mL of DI water was added, and the mixture was boiled for another 5 min and cooled to room temperature to form a TiO_2_ sol. The TiO_2_ sol was added into a prepared composite silica sol-gel to form a SiO_2_-TiO_2_ sol-gel with TiO_2_ content of 2.3 wt%.

### 2.3. Fabrication of Closed-Surface Self-Cleaning ARCs

PV glass was washed with commercial detergents, followed by ultrasonic cleaning in DI water for 15 min and then flushing with EtOH. The cleaned glass was dipped in AR sol and rinsed for 1 min at room temperature; then, it was lifted at 3.3 mm/s to form a thin film on the glass. Organics, including the PAA cores, were burnt away via calcination in a muffle furnace at 500 °C for 2.5 h. After calcination, a closed-surface self-cleaning ARC was formed on the glass. 

### 2.4. Characterization of Microstructure and Morphology

The morphology of the ARC was measured by field emission scanning electron microscopy (FESEM), where cross-sections of ARC samples were prepared by mechanically breaking the coated PV glasses after calcination. The microstructure of the sol-gel was investigated using a JEM-2100 transmission electron microscope (TEM), JEOL, Tokyo, Japan, with an acceleration voltage of 150 kV. Sol-gel samples were first diluted in EtOH and then added onto a carbon-coated copper grid for TEM observations. Nanoscopy IIIa scanning probe atomic force microscopy (AFM) (Digital Instruments, Santa Barbara, CA, USA) was used to measure the roughness of the thin films.

### 2.5. Evaluation of Photocatalytic Properties 

SiO_2_-TiO_2_ ARC-coated glasses and bare glasses were immersed in a 2 mg/L methylene blue (MB) aqueous solution. These samples were then irradiated under a 120 W UV lamp, and the transmission curve was measured after different UV exposure times.

### 2.6. Optical Property Measurements

Since the crystalline silicon solar cell’s bandgap is 1.12 eV, only wavelengths from 300 to 1100 nm were within its spectral response range [20]. The transmission spectra in this range were measured by a PerkinElmer Lambda 750S UV-vis-NIR spectrophotometer (Waltham, MA, USA), with a scanning step of 5 nm. The coating thickness and RI of the ARCs were measured by a Semilab SE 1000 ellipsometer (Budapest, Hungary).

### 2.7. Flashing Test and External Quantum Efficiency (EQE) Measurement of a PV Module

Solar cell parameters, like cell efficiency, open-circuit voltage, and short-circuit current, were measured by performing flashing tests under standard test conditions (STC, 25 °C, 1000 W/m^2^) with a Halm cell tester. The grade of the AM1.5 irradiance spectra was no less than AAA, which was generated from a Xe bulb. Based on the AM1.5 irradiance spectra (IEC60904), a short-circuit current could also be calculated from the cell and module’s EQE curve. The EQE curve is the spectral quantum response of a solar cell, which was determined by a Bentham PVE300 (Bentham Instruments Ltd., Berkshire, UK) used on a mono-crystalline solar cell, both with and without lamination, in the wavelength range 300–1100 nm.

### 2.8. Long-Term Reliability Evaluation

Damp heat tests (85 °C and 85% relative humidity) were conducted to evaluate the reliability performance of the ARCs. Glasses coated with an ARC were kept in a climate chamber for 1000 h or longer with damp heat conditions. No visual defects were allowed after the damp heat test, and the transmittance of glass was measured both before and after the damp heat tests, normalized transmittance degradation (transmittance after damp heat/initial transmittance) was used to assess the reliability of the ARC. Here, damp heat tests were conducted in an Espec AR-series climate chamber.

## 3. Results and Discussion

### 3.1. Structure and Morphology of SiO_2_-TiO_2_ Closed-Surface ARC

**Figure 1:**
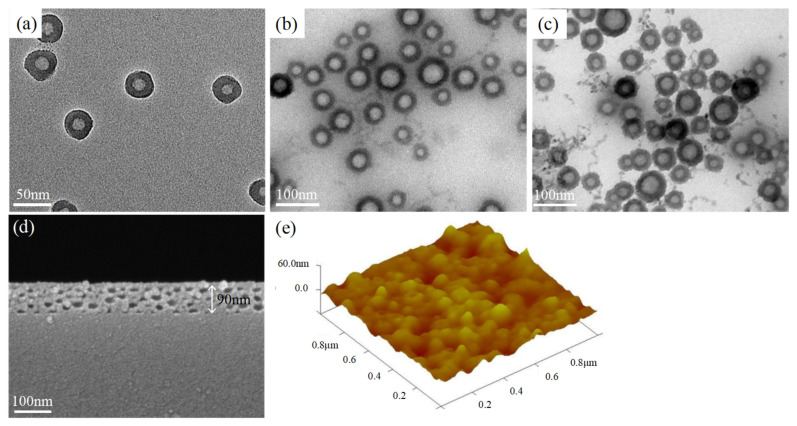
(**a**) Transmission electron microscope (TEM) image of silica hollow nanospheres (HNs), (**b**) TEM image of silica HNs/acid-catalyzed silica, (**c**) TEM image of silica HNs/acid-catalyzed silica/TiO_2_ nanoparticles, (**d**) cross-section image of closed-surface antireflective coating (ARC), and (**e**) Air force microscopy image of the surface roughness.

Multiple works [21,22] have synthesized silica HN solutions and formed high-porosity silica ARCs. Here, PAA was first added to ethanol under vigorous magnetic stirring to form monodisperse nanoscale PAA spheres, which were subsequently wrapped by silica via hydrolysis with TEOS. The size of the PAA spheres was modulated by the concentration of PAA in ethanol and stirring speed, while the TEOS dosage was used to control the silica shell thickness. The PAA cores were removed during calcination at 500 °C. In this paper, silica HNs with a PAA core diameter of 40–50 nm and a silica shell thickness of 8–10 nm was prepared, as shown in Figure 1a. 

ARC made from solo silica HNs had poor adhesion to substrate and was not durable enough for outdoor applications [23]. On the contrast, the silica ARCs made from acid-catalyzed silica sol-gel possess a low porosity, resulting in a high refractive index [24], while acid-catalyzed silica has a linear or random branch-chain structure, which strongly bonds to glass substrates [25]. Here, silica HNs were dispersed into a mixture of TEOS, H_2_O, HCl, and EtOH, and then branched acid-catalyzed silica was grown on the silica HNs, as shown in Figure 1b. The branch-chain silica was used to fill the spaces between HNs in the ARC. The KH-560 was a key factor to prepare a closed-surface ARC, it first produced silanol groups through hydrolysis reaction with water, then followed by partial condensation to form oligomers, the silanol groups were then hydrogen bonded to silica surface [26], thus acted as a binder which was able to link individual particles by covalent bonds, increasing the bonding strength of particle-to-particle, as well as particle-to-substrate. TiO_2_ nanoparticles obtained from hydrolysis were also added into this nanocomposited silica sol-gel under stirring, as shown in Figure 1c. By precisely controlling the ratio of HNs, acid-catalyzed silica, and TiO_2_, a closed-surface self-cleaning ARC with an RI of approximately 1.30 and TiO_2_ content of around 2.3 wt% was obtained.

Figure 1d shows a cross-section of the ARC on PV glass. The hollow spheres were densely packed, with no voids on the top surface. Inside the thin film, silica from the acid-catalyzed sol-gel filled all spaces between HNs and acted as a binder for the HNs. Very few collapsed HNs were found, which means 8–10 nm shells are strong enough to sustain the structure of the hollow spheres. The interface between the glass and ARC was fully covered by silica, either by the HN shell or silica from the acid-catalyzed silica, indicating a high binding strength of the ARC and glass substrate. AFM measurements showed that the mean-square roughness of the ARC was 20–23 nm. As shown in Figure 1e, this low surface roughness is good for anti-abrasion.

### 3.2. Photocatalytic Properties of SiO_2_-TiO_2_ Closed-Surface ARCs

**Figure 2:**
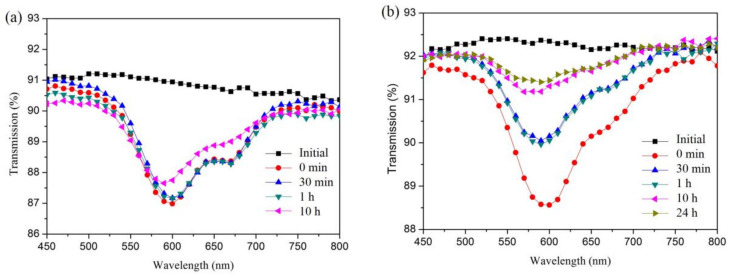
Transmission curves of glass post MB dipping under different UV soaking times: (**a**) bare glass, (**b**) SiO_2_-TiO_2_ closed-surface ARC glass.

TiO_2_ nanoparticles usually possess photocatalytic properties because of their higher electron-hole pairs delivery ability, larger specific surface area, and higher surface permeability, which allow the excited electron-hole pairs to diffuse toward the surface before their recombination [15,16,18,19]. Forming transparent TiO_2_ nanocomposite thin films on various substrates is a practical application for organic contaminants removal. The photocatalytic activity of SiO_2_-TiO_2_ closed-surface coating was studied by its decomposition of MB. Several SiO_2_-TiO_2_ ARC-coated glasses, together with bare glasses, were immersed into MB solutions; these samples were then irradiated under a 120 W UV lamps. After 5 days of irradiation, the MB solution with SiO_2_-TiO_2_ ARC-coated glasses became transparent, while the bare glass sample stayed blue. The photocatalyst activity could also be observed directly on the SiO_2_-TiO_2_ coatings; after lifting the SiO_2_-TiO_2_ closed-surface AR glass from the MB solution, the glass transmittance showed a significant decrease, and a trough at the wavelength of around 600 nm could be observed in the transmission curve, which was an indicator of MB absorbing. The transmittance of 600 nm increased as a function of irradiation time under UV dosing. In addition, the trough was completely gone after 24 h of irradiation, as shown in Figure 2, which means that the MB was completely decomposed. Therefore, the SiO_2_-TiO_2_ closed-surface ARC possessed excellent photocatalytic activities. 

### 3.3. Optics of SiO_2_-TiO_2_ Closed-Surface ARCs

**Figure 3:**
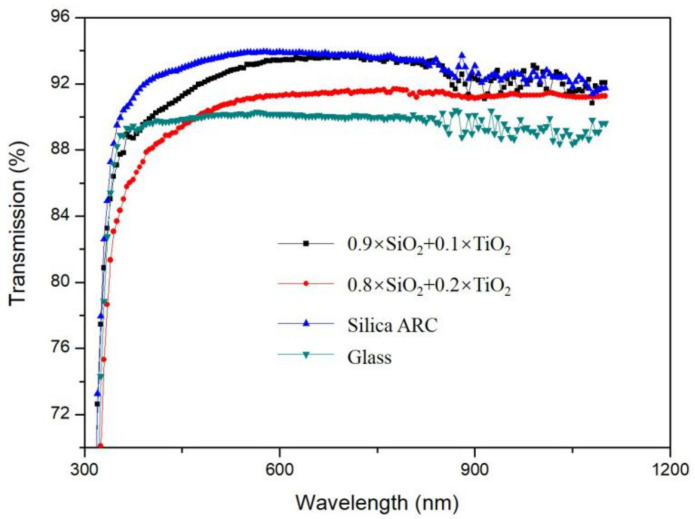
Transmission curves of ARCs with different TiO_2_ contents.

There are two critical indexes of ARC optical properties: refractive index and reflectivity. Usually, the RI was controlled by the porosity of coating and the reflectivity was dominated by the thickness of ARC and partly influenced by the RI. Thickness of ARC decided the wavelength of peak value in transmission curve, which should be around 600 nm to obtain the highest irradiation of solar light. According to previous study [13], the withdrawal speed of glass from the sol was set to 1.0mm/s to have the maximum transmittance at wavelength of 600 nm for PV modules to have receive high solar irradiation. The RI of a TiO_2_ thin film was about 1.8 if the glass substrate is coated by pure TiO_2_ nanoparticles [27,28]. This RI is much higher than the target RI of 1.23 for ARCs on glass substrate. On the other hand, by tuning the cavity of silica ARC, the RI of silica coating could be manipulated varying from 1.1 to 1.5 [29], so by mixing TiO_2_ nanoparticals into silica coating is a promising way to control the RI of TiO_2_ nanocomposite coating. In this study, a nanocomposite sol-gel containing HNs and acid-catalyzed silica was first prepared, which resulted in a closed-surface silica ARC with an RI of around 1.25–1.27. Second, the TiO_2_ nanoparticles were gradually added into the silica sol-gel via stirring, and an MB decomposition test was conducted to determine the minimum TiO_2_ content for the ARC to display a self-cleaning function. Figure 3 shows the transmission curves of different TiO_2_ contents. The peak value of the transmission curve moved downwards upon the increasing in TiO_2_ content, and the wavelength of peak value also moved slightly to infra end, because the RI of coating increased with higher TiO_2_. After optimization, an ARC RI of 1.30 was obtained to have both closed-surface structure and self-cleaning function, and the transmittance reached 94.03% on single side ARC coating glass samples. The HN diameter range was 40–50 nm with a silica shell thickness of around 8–10 nm, and TiO_2_ nanoparticle content was of about 2.3 wt% in the nanocomposite sol-gel. Compared with the glass substrates, the transmission was greatly enhanced in the visible-to-IR range on the coated glass, with a weighted transmittance that was around 3.01% higher than that of the glass substrate.

### 3.4. Photovoltaic Device Performance of SiO_2_-TiO_2_ Closed-Surface ARC

The short-circuit current density (*J_sc_*) of a solar cell or PV module can be calculated using Equation (1) [30]:

(1) $$J_{SC}=q\int_{\lambda }F\left(\lambda \right)\cdot EQE\left(\lambda \right)d\lambda =q\int_{\lambda }F\left(\lambda \right)\cdot IQE\left(\lambda \right)\cdot \left[1-R\left(\lambda \right)\right]d\lambda$$

where *λ* is the wavelength, *q* is the electron charge, *F(λ)* is the photon flux, *EQE(λ)* is the external quantum efficiency, *IQE(λ)* is the internal quantum efficiency, and *R(λ)* is the reflectance. By applying an ARC on glass, the *R(λ)* of a solar module is reduced, which increases the *J_sc_*.

A commercial 15.8 × 15.8 cm^2^ monocrystalline Si solar cell was selected in this experiment. The short-circuit current and the spectral response of each cell were measured. Cells with similar electrical parameters were laminated into modules to measure the EQE of each module. A typical structure of a glass/ethylene vinyl acetate (EVA)/cell/EVA/backsheet was used in these modules, where the backsheet was a composite foil on the backside of the PV modules to protect the cells from moisture and humidity. EVA stands for ethylene vinyl acetate encapsulant foil. In the case of the PV module, only the airside of the PV glass could be coated by the ARC because the use of an interior ARC, due to the introduction of an optical mismatch layer between the glass and the EVA foil, negatively impacts the light transmission into the cell, as explained in [31]. 

**Figure 4:**
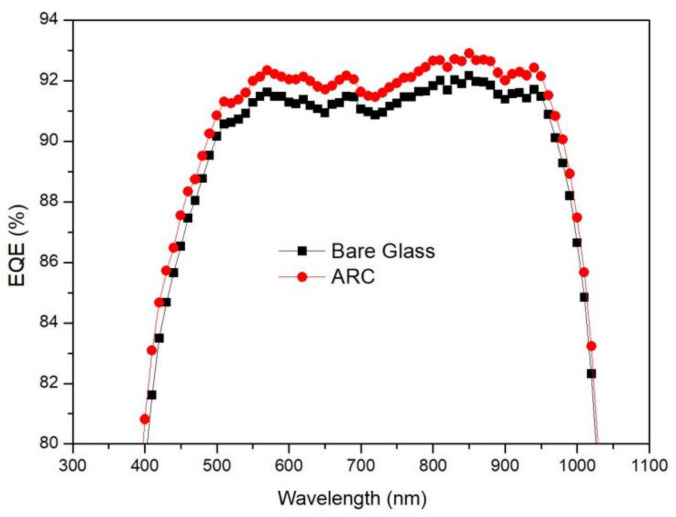
External quantum efficiency (EQE) curves of typical modules encapsulated with closed-surface SiO_2_-TiO_2_ ARC glass compared with capsulation by bare glass.

The EQE curves of typical modules encapsulated with SiO_2_-TiO_2_ closed-surface ARC-coated glass are shown in Figure 4. Compared to capsulation with bare glass, the quantum efficiency of the module with an ARC was greatly enhanced in the wavelength range 400–1000 nm, especially 600–800 nm, which are the wavelength ranges of the highest solar irradiance. This EQE improvement is consistent with the improved transmittance. Compared with the short-circuit current densities calculated from the EQE curves, the *J_sc_* of the module with an ARC was 2.14% higher than that of the module with bare glass. On the same samples, the *J_sc_* gain of the module from IV measurements was about 2.32%. Considering the measurement error of different characterization methods, these two *J_sc_* improvements are considerably aligned.

### 3.5. Mechanical Properties of SiO_2_-TiO_2_ Closed-Surface ARC

Many works have revealed that TiO_2_ can increase the robustness of silica thin films [32,33]. To evaluate the final mechanical properties of the SiO_2_-TiO_2_ closed-surface ARC, three different tests were conducted. First, the adhesion between the ARC and glass was evaluated. The 3M scotch transparent tape was pressed on the ARC and then peeled-off quickly (ASTM D3359: cross-cut/Tape test). No thin film peeled off from the glass substrate, indicating strong bonding between the ARC and glass. Second, the abrasion resistance was measured with the falling sand abrasion test (ASTM D896). Test samples were placed with a 45° angle at the bottom, and a 1000 mm tube was placed vertically on top of the samples; then, standard sand fell onto the test samples along the tube. The SiO_2_-TiO_2_ closed-surface ARC could endure 63 L of sand falling before the coating was removed, meaning that it is better than silica ARC, which only endured 50 L sand falling in a comparison test. Third, the thin-film hardness was evaluated via a pencil test (ASTM D3363-92a), in which the hardest pencil that leaves no visible trace on the ARC defined the final pencil hardness. The ARCs demonstrated a 4H pencil hardness. All three tests showed that the SiO_2_-TiO_2_ closed-surface ARC had good mechanical properties.

### 3.6. Long-Term Reliability of SiO_2_-TiO_2_ Closed-Surface ARC

For PV modules and windows, the ARCs should undergo little degradation for decades in the field; therefore, ARCs applied on glass must be able to survive severe accelerated aging tests, especially those at high temperatures and humidity. According to IEC 61215 [34], the power degradation of PV modules must be less than 5% after 1000 h of the damp heat (DH: 85 °C and 85% relative humidity) test. This IEC standard is also widely used to evaluate the durability of ARCs. According to previous work [35], during damp heat tests, the H^+^ of water infiltrates the thin film via voids and then exchanges with cations to form a hydrated layer. Subsequently, the Si-O-Si bridging bonds, both in the ARC and between the coating and substrate, are gradually hydrated, and the silica network is degraded. Since other materials in the modules also suffer degradation during aging tests, a transmittance degradation less than 3% after DH 1000 h is normally required for an ARC [36]. Previously, silica closed-surface ARCs were prepared to minimize the degradation of an ARC [13]. As it had almost no voids on its surface, the aqueous solution had very few infiltration paths. In this study, glasses with a single side SiO_2_-TiO_2_ closed-surface ARC (T% = 94.00%), together with control samples with a single-side silica closed-surface ARC (T% = 94.35%), were placed into a DH chamber for 1000 and 2000 h.

**Figure 5:**
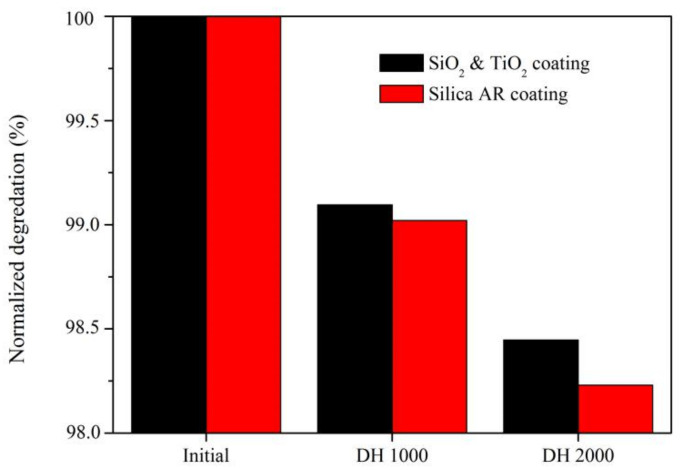
Normalized transmittance of ARCs after damp tests.

The degradation of the transmittance is presented in Figure 5. The transmittance of the SiO_2_-TiO_2_ closed-surface ARC decreased to 93.15% after DH 1000 h and 92.54% after DH 2000 h. Normalized transmittance were 99.10% and 98.45%, respectively, the degradation were around 0.90% and 1.55%, which were far less than 3%. In comparison, the normalized transmittance of the single-side silica closed-surface ARC was 99.02% and 98.23% after DH 1000 h and DH 2000 h, degradation was also within 3%. Both types of ARCs have excellent resistance to high moisture contents and high temperatures, and adding TiO_2_ nanoparticles did not reduce the reliability of the ARC.

## 4. Conclusions

A one-dipping process was used to fabricate a SiO_2_-TiO_2_ closed-surface ARC. The sol-gel was prepared by adding TiO_2_ nanoparticles into a mixed nanosilica sol-gel that contained branch-chain-structured, acid-catalyzed silica grown on hollow silica spheres. A transmittance of up to 94.03% was obtained when a single side of PV glass was coated with the ARC. No voids were found on the front surface of this ARC during surface morphology tests, which allowed it to prevent moisture absorption. The mechanical properties, which were measured in tape tests, abrasion resistance tests, and pencil hardness tests, showed that this ARC had good robustness. The SiO_2_-TiO_2_ closed-surface ARC showed excellent reliability in high-moisture and high-temperature conditions. Compared with bare glass, a monocrystalline silicon PV module laminated with ARC-coated glass showed a 2.14–2.32% gain in short-circuit current during IV measurements, which was confirmed by the EQE tests. This one-dip SiO_2_-TiO_2_ closed-surface ARC also possessed photocatalytic activity. It has great potential applications for PV modules located in extreme climates.

## Data Availability

The raw/processed data required to reproduce these findings cannot be shared at this time due to legal or ethical reasons.
